# The Impact of COVID-19 on Essential Medicines and Personal Protective Equipment Availability and Prices in Saudi Arabia

**DOI:** 10.3390/healthcare9030290

**Published:** 2021-03-07

**Authors:** Rana Aljadeed, Yazed AlRuthia, Bander Balkhi, Ibrahim Sales, Monira Alwhaibi, Omar Almohammed, Abdulaziz J. Alotaibi, Ali M. Alrumaih, Yousif Asiri

**Affiliations:** 1 Department of Clinical Pharmacy, College of Pharmacy, King Saud University, Riyadh 11451, Saudi Arabia; raljadeed@KSU.EDU.SA (R.A.); bbalkhi@KSU.EDU.SA (B.B.); isales@ksu.edu.sa (I.S.); malwhaibi@ksu.edu.sa (M.A.); oalmohammed@ksu.edu.sa (O.A.); yasiri@ksu.edu.sa (Y.A.); 2 Pharmacoeconomics Research Unit, College of Pharmacy, King Saud University, Riyadh 11451, Saudi Arabia; 3 Saudi Medical Supply Chain Assembly, Saudi Pharmaceutical Society, Riyadh 11451, Saudi Arabia; Sulais99@gmail.com; 4 Pharmaceutical Care Department, Medical Services for Armed Forces, Ministry of Defense, Riyadh 11451, Saudi Arabia; rumaiham@gmail.com

**Keywords:** COVID-19, drug shortage, healthcare, supply chain, Saudi Arabia

## Abstract

This was a questionnaire-based cross-sectional study that explored the impact of the COVID-19 pandemic on the availability of essential medicine and personal protective equipment (PPE) in Saudi Arabia. Purposive sampling technique was used to recruit individuals working in the supply chain departments in different healthcare sectors in Saudi Arabia. One hundred and three pharmaceutical and medical supply chain employees participated in the study. Most of the participants (58.3%) were aged ≥35 years, male (65%), and pharmacists (92.2%). The majority of participants had at least two years of experience in supply chain (77.6%), worked in public hospitals (95.15%), and were mostly working at healthcare institutions located in Riyadh province (59.2%). Approximately 51% of the participants reported shortages of 10 or more essential drugs. Tocilizumab, hydroxychloroquine, lopinavir/ritonavir, ribavirin, dexamethasone, enoxaparin, interferon beta-1b, cisatracurium besylate, prednisolone, hydrocortisone, methimazole, and methylprednisolone were reported to be in shortage by at least 8% of the participants. Almost 70% of the participants reported that the pandemic did not significantly impact the prices of prescription drugs in shortage (e.g., ≥25%). Moreover, about 70% of the participants reported direct purchasing or procurement of drugs in shortage. Surgical masks, face shields, medical gowns, and N95 respirators were reported to be in short supply by 33% or more of the participants. Approximately 53% of the participants reported the prices of PPE in shortage had seen an increase by at least 25% during the pandemic. Although the COVID-19 pandemic has caused a significant disruption in the global pharmaceutical supply chain, its impact was largely manageable in Saudi healthcare institutions. This can be attributable to multiple reasons such as the effective exchange programs between hospitals and the drastic increase in public healthcare spending to ameliorate the negative impact of the pandemic on the healthcare sector.

## 1. Introduction

In March 2020, the World Health Organization (WHO) announced that the coronavirus (COVID-19) outbreak had become a global pandemic [1]. As of 11 December 2020, the virus has infected more than 72 million individuals and caused over 1.61 million deaths worldwide [1]. The pandemic has greatly impacted the global economy, especially the pharmaceutical sector [2]. With each passing day, the medical supply chain struggles to meet the increasing demand on highly needed pharmaceuticals and personal protective equipment (PPE). This pandemic has exposed the fragile medical supply chains which are largely dependent on imports of active pharmaceutical ingredients (APIs) from China and India [3,4]. It is believed that pharmaceutical companies in the United States (U.S.) import nearly 80% of their APIs needs from India and China, the world’s top API suppliers [4]. As China was the first country to be severely impacted by COVID-19, the three-month lockdown has significantly curtailed the medical supply chain flow, leaving a lasting effect on the global API market [3].

To aid in addressing this unprecedented situation, regulatory authorities, most notably the United States Food and Drug Administration (FDA) and American Society of Health-System Pharmacists (ASHP), have published a “drug shortage list” consisting of potential COVID-19 treatments and intensive care unit (ICU) medications for COVID-19 patients requiring mechanical ventilation [5,6]. The FDA released a list of drugs in short supply, which included furosemide injections, dopamine, dobutamine, fentanyl, morphine, heparin, propofol, midazolam, and dexmedetomidine [6]. Overall, in a six-month period, this year’s drug shortages have been equivalent to 87% of the shortages reported for the entire year in 2019 [7]. This level of drug shortages is projected to escalate further as COVID-19 cases rise [7]. The ASHP also published their list of over 200 drugs currently in shortage [5]. Their list included sedatives, neuromuscular blocking agents, and cardiovascular drugs in addition to potential COVID-19 treatments such as hydroxychloroquine (HCQ) and chloroquine (CQ) [5]. The HCQ and CQ shortages are particularly serious because these drugs are also used to treat autoimmune diseases, such as rheumatoid arthritis [7]. Although the shortages of HCQ and CQ have been addressed swiftly, shortages of other essential drugs, such as dexamethasone, have been reported [7].

The COVID-19 pandemic has also affected the supply chain of highly needed PPE [8]. The pandemic has significantly increased the demand for protective goggles, medical masks, protective gowns and gloves and exacerbated the already strained PPE supply chains, leading to shortages of numerous PPE [8]. Therefore, the ASHP began to survey members of the Section of Pharmacy Practice Leaders (SPPL) in order to obtain real-time status of the U.S. pharmacy resources including PPE and ICU medications during the pandemic and publish the results biweekly [9]. Based on the ASHP published reports, shortages of surgical masks, N95 respirators, and other vital supplies for healthcare settings have been reported [9]. Moreover, critical ICU drugs, such as cisatracurium, fentanyl, ketamine, midazolam, and vecuronium have been reported to be in short supply [9].

In Saudi Arabia, drug shortages have been frequently reported over the past five years due to a multitude of reasons [10,11,12,13]. However, this pandemic has exacerbated the situation both locally and globally [14]. Few studies have described and characterized the status of Saudi Arabia’s drug shortages before the pandemic [10,11,12,13]. Failure to inform the Saudi Food and Drug Authority (SFDA) of drug shortages by the different healthcare entities, poor supply chain management, poor enforcement of regulations by the ministry of health, reliance on prescription drug imports since the local manufacturers only cover 20–25% of the domestic drug consumption, low profit margin of some old and essential drugs, and inflexible traditional public procurement policy were believed to be the main reasons behind the frequently witnessed drug shortages [10]. Although the SFDA pharmaceutical pricing policy ensures a reasonable profit margin for pharmaceuticals especially essential drugs by choosing a reasonable price based on a basket of reference countries [15], this profit margin is significantly eroded in the tendering process (i.e., the price can be 70% lower or more), which is largely centralized through National Unified Procurement Company for Medical Supplies (NUPCO) in Saudi Arabia [16]. NUPCO centralizes the procurement, warehousing, and distribution of medical equipment and supplies in addition to pharmaceuticals by negotiating with leading medical companies and manufacturers and selecting one bidder at the end with the lowest price [16]. This is believed to be partly responsible for the drug shortages witnessed over the past five years [10,13]. Therefore, if shortages occur, the healthcare institutions in general, and the public ones in particular, resort to directly purchasing any drug in shortage from the market based on SFDA prices, which are significantly higher than the tender prices. Additionally, the withdrawals of multiple pharmaceutical products by the SFDA due to failure of the manufacturers of these products to comply with the Good Manufacturing Practices (GMP) have also contributed to the drug shortages in Saudi Arabia [17,18]. However, this pandemic has affected the global medical supply chain and introduced serious challenges to the quality of care both locally and internationally. Therefore, we aimed to explore the status of essential drug and PPE shortages during the COVID-19 pandemic in different healthcare sectors from the perspective of medical supply chain employees who are involved in the procurement planning and purchasing of pharmaceuticals and medical supplies in Saudi Arabia.

## 2. Methods

This was a questionnaire-based cross-sectional study that explored the status of drug and PPE shortages among employees in the supply chain management departments of different healthcare institutions in Saudi Arabia. For this study, shortages were defined as the unavailability of any drug or PPE for two weeks or more in any healthcare institution due to the inability of the manufacturer or licensed importer to supply the market with the needed quantities to meet the current demand [10]. In order to explore the status of drug and PPE shortages from the perspective of the employees in the medical supply chains departments, a 13-item questionnaire was developed (Appendix A). The questionnaire included questions about age, gender, profession, place of work, geographic region, years of experience in the supply chain, frequency of drug shortages, the names of drugs in short supply, in general, and the essential drugs based on the World Health Organization (WHO) list of essential medicines, in particular, as well as other drugs used in the management of COVID-19, names of PPE in short supply [19], whether a direct purchase order (e.g., purchasing of pharmaceuticals and/or medical supplies from specified pharmaceutical or medical supply companies or wholesalers outside the scope of the original contract) was placed for drugs and/or PPE in shortage during the pandemic, and the difference in drug and PPE prices before the pandemic and now. Purposive sampling was utilized to recruit participants working in the medical supply chain departments. Thus, a list of 174 employees in the medical supply chain departments in different healthcare sectors (e.g., public hospitals, private hospitals, university-affiliated hospitals, etc.) was identified using the contact database of the Saudi Healthcare Supply Chains Assembly in the Saudi Pharmaceutical Society. The list included senior employees in the healthcare affairs of all ministries that provide healthcare services to their employees as well as the heads of pharmacy planning and purchasing in the main tertiary care hospitals in Saudi Arabia. The questionnaire was emailed to the potential participants using Google^®^ forms, and three email reminders were sent to those who did not fill out the questionnaire. Participation was voluntary and the participants did not receive any financial or non-financial incentives. The study was approved by the Ethics Review Board Committee of the Central Ministry of Health (20–152E) in Riyadh, Saudi Arabia, and was conducted from 24 July to 30 September 2020. Descriptive statistics using Chi-square and Fisher’s exact tests were performed, and the results were presented as frequencies and percentages. All statistical analyses were conducted using SAS^®^ version 9.4 (SAS^®^ Institute Inc., Cary, NC, USA).

## 3. Results

**Figure 1:**
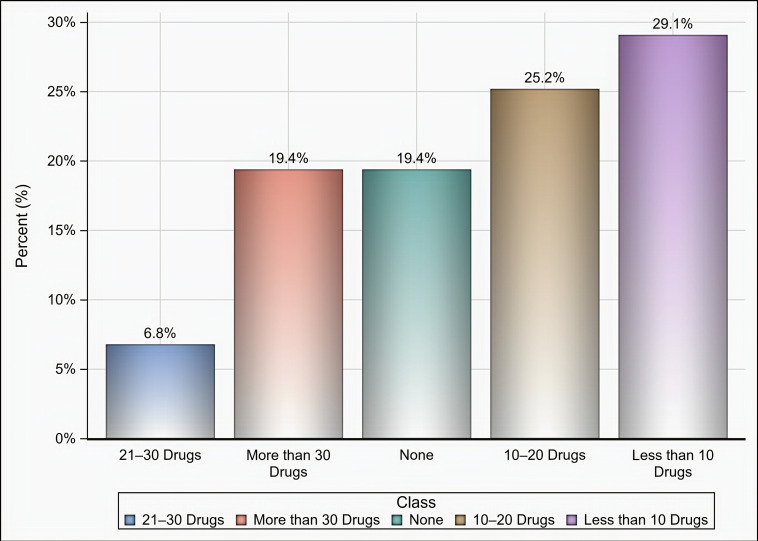
The number of drugs in shortage based on participants’ responses.

**Figure 2:**
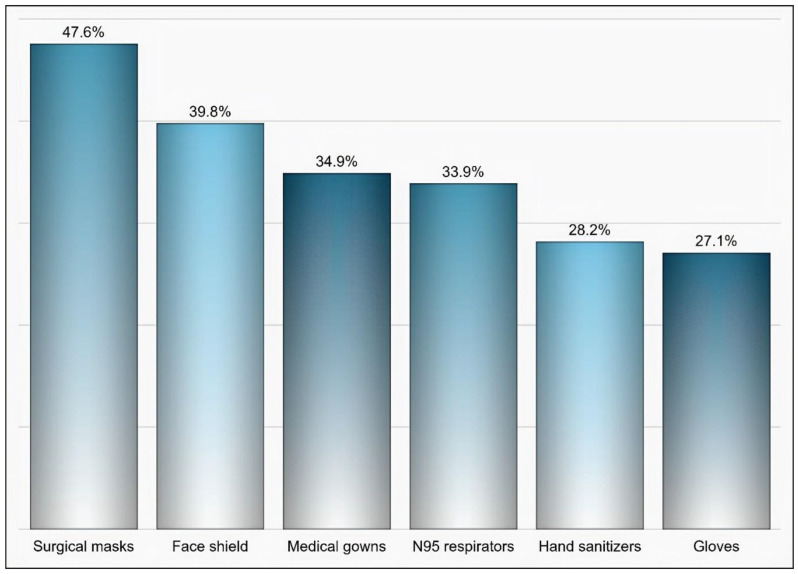
The personal protective equipment (PPE) that was reported to be in shortage based on participants’ responses.

**Figure 3:**
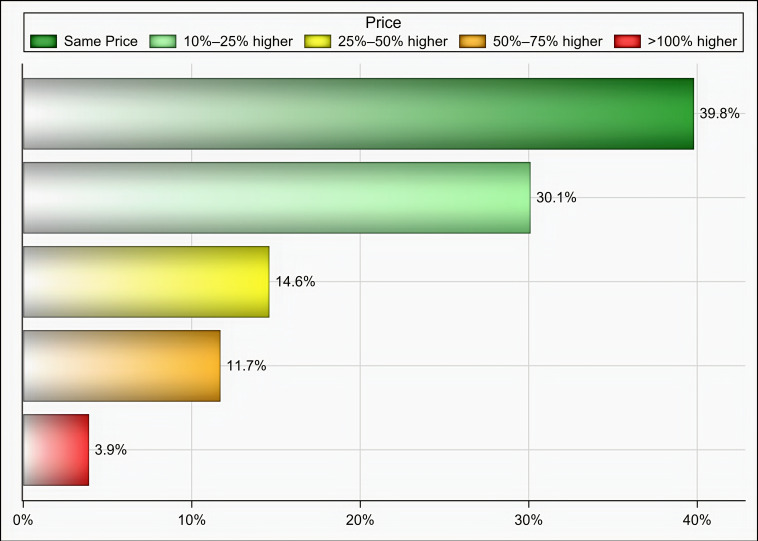
The impact of COVID-19 on prices of drugs in short supply based on participants’ responses.

**Figure 4:**
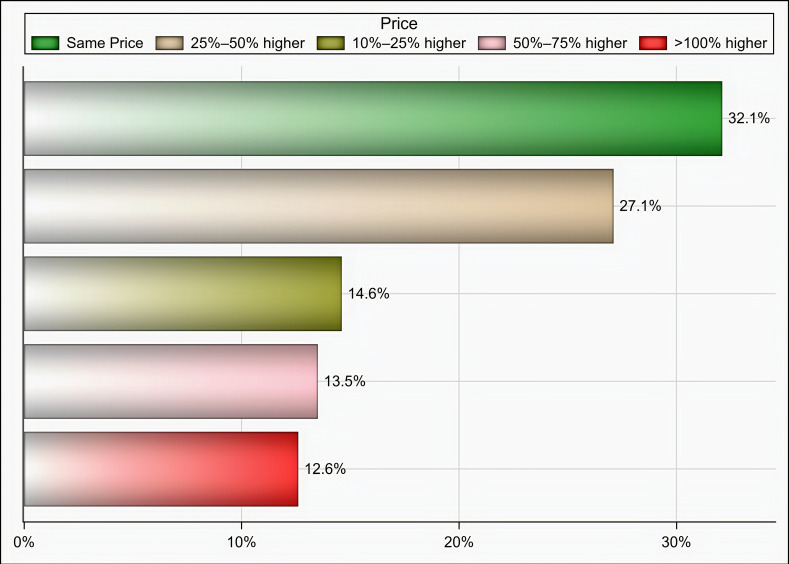
The impact of COVID-19 on prices of PPE in short supply based on participants’ responses.

Out of 174 individuals who were sent an invitation to participate, 103 (59.19%) completed the questionnaire. Most of the participants were male (65%), aged 35 years and above (58.25%), pharmacists (92.2%), had at least two years of experience in medical supply chains management (77.67%), located in Riyadh (59.22%), and affiliated with the ministry of health (41.75%) and ministry of defense and aviation health affairs (24.27%) (Table 1). Approximately 51% if the participants reported that their healthcare institutions had 10 or more drug shortages in comparison to 29.1% and 19.4% of participants who reported fewer than 10 drug shortages and no drug shortage, respectively, (*p* < 0.001) as shown in Figure 1. Tocilizumab, hydroxychloroquine, lopinavir/ritonavir, ribavirin, dexamethasone, enoxaparin, interferon beta-1b, cisatracurium besylate, furosemide, prednisolone, hydrocortisone, methimazole, and methylprednisolone were the most commonly reported drug shortages by at least 8% of the participants as shown in Table 2. With regard to the PPE in shortage, surgical masks, face shields, medical gowns, N95 respirators, hand sanitizers, and gloves were reported to be in shortage by at least 27% of the participants (*p* < 0.001) as shown in Figure 2. More than two-thirds (69.9%) of the participants reported that direct purchase orders were placed during the COVID-19 pandemic due to the shortages of both drugs and PPE that could not be addressed by the local exchange programs between the different healthcare institutions. Only 30.2% of the participants reported that prices of drugs in shortage have increased by 25% or more during this pandemic (*p* <0.001) as shown in Figure 3. On the other hand, 53.2% of the participants reported that the prices of PPE in shortage have increased 25% or more during the COVID-19 pandemic (*p* <0.001) as shown in Figure 4.

## 4. Discussion

To our knowledge, this is the first published study assessing the impact of the COVID-19 pandemic on essential drugs and PPE shortages in Saudi Arabia. The results indicated that the global disruptions in the supply chains had profound effects that ricocheted throughout many countries including Saudi Arabia [1,2,3,4]. Moreover, this pandemic highlighted several shortcomings in the Saudi drug and medical equipment supply chain, such as, the poor communication between the different public and private health institutions and the SFDA about the status of drug shortages in these institutions, overreliance on drug imports, and failure of the local drug manufacturers to meet the local need of essential drugs and medical supplies [10]. While the direct impact of these shortages on the quality of care and patient outcomes is still unknown, previous studies have reported an increase in mortality, adverse reactions, medication errors, and hospitalizations [20,21,22,23,24,25,26].

Tocilizumab, hydroxychloroquine, lopinavir/ritonavir, ribavirin, dexamethasone, enoxaparin, interferon beta-1b, cisatracurium besylate, furosemide, prednisolone, hydrocortisone, methimazole, and methylprednisolone were the most common essential medications mentioned by respondents as being difficult to obtain. This list includes many of the medications listed on ASHP’s Drug Shortage List, which highlights the global ramifications of the supply chain shortages [5]. As of December 2020, hydroxychloroquine, dexamethasone, enoxaparin, cisatracurium besylate, and furosemide remain on the drug shortage list [5]. Additionally, previous lists have included prednisolone, hydrocortisone, and methylprednisolone [5].

Nearly 30% of participants indicated that there were inadequate supplies of PPE. Similar to essential medications, PPE shortages have been reported in the United States (US), European Union (EU), and globally in varying degrees [5,27,28]. The reported shortage of PPE in this study is consistent with previously published studies that reported shortages of PPE and the higher risk of infection and transmission associated with these shortages [29,30]. In addition, lack of access to PPE in Europe has been attributed to the increased rates of COVID-19 exposure and mortality in first responders [29]. Healthcare workers in Saudi Arabia have reported a moderate level of concern regarding COVID-19, and adequate PPE supplies have been deemed highly important due to the high contagiousness and rapid spread of COVID-19 infection [29]. Additionally, the shortage of essential PPE led to reuse of disposable PPE, putting the life of frontline healthcare workers at risk of catching contagious infections including COVID-19 and spreading different contagious infections to patients and the public [30].

Furthermore, approximately 70% of participants indicating that direct purchase orders were placed during the pandemic for medications and PPE in essence is a direct reflection of the impact of the pandemic upon the supply chain. Direct purchases are processed through NUPCO and it has been previously implicated as the source of drug shortages [11,12,13]. Most of the time, pharmaceutical or medical supply companies that win the contracts in the tendering process are those that offered the lowest prices. This can lead to a drop in the number of companies winning contracts over time, and may force some companies to leave the market which can eventually lead to drug and medical supply shortages mainly due the inability of the contract winners to meet the market demand, especially during unprecedented times such as the case with the COVID-19 pandemic [10]. However, these drug shortages have occurred on an international scale and are expected due to the nature of the crisis [20]. The large majority of participants requesting direct purchases also indicates poor medication and PPE planning and stocking for emergencies, such as the COVID-19 pandemic or any disruption of essential drug supply chains [11]. The differences in the increases in the prices of medications and PPE is also intriguing. Variations in medication price increases could be attributed to the differences in treatment strategies implemented in each healthcare system due to the rapidly changing recommendations and new potential therapies [31]. PPE price increases were reported by over half of the respondents. The cause of this discrepancy among the respondents is unknown and requires further investigation. Moreover, the importance of demand planning and forecasting for pharmaceuticals and medical supplies cannot be emphasized enough to enhance the efficient utilization of resources.

Overall, the procurement challenges appeared to be stabilized through the centralized purchasing agreements negotiated by NUPCO. NUPCO was able to seamlessly conclude deals for the acquisition of vital equipment such as COVID-19 testing kits to expand testing throughout the country early during the pandemic [32]. Likewise, the Joint Procurement Agreement (JPA) in the EU was also effective in providing essential medications and equipment to member states quickly and efficiently and preventing price gouging and drug and supply hoarding [27]. Moreover, incidents such as taking a complete monopoly of the July 2020 production of remdesivir adversely affected global supply chains [32,33,34]. Furthermore, the capitalistic dogma has led to both federal versus state as well as intrastate competition for essential medications and equipment. With the heightened demand in the United States for products, the law of supply versus demand has led to price inflation.

In the midst of these shortages, several strategies have been proposed in order to curtail current and future incidents from occurring. Implementation of policy changes, such as penalties to prevent price gouging, and mandating that procurement data is fully disclosed to the public may be effective methods for countries to consider [35]. In addition, an organized public platform in order to search for items provided by multiple suppliers and also establishing public organizations to monitor governmental policies and transactions may increase citizen confidence during the crisis. Policymakers can establish warning systems to preemptively inform concerned parties of expected shortages, galvanize local manufacturers to begin production of drugs that may be in high demand and low in supply, creating a national stockpile for essential medications, and facilitate better means of communication among all stakeholders [11,36,37]. Additionally, establishing a medication exchange and sharing a network program between the regional countries, such as the Gulf Cooperation Council member states, should be encouraged. Further, utilizing pharmacists for selecting appropriate evidence-based alternative therapies and prioritizing the most suitable candidates for therapy during shortages [20,38]. In addition, the creation of guidelines for appropriate use, algorithms, medication alerts, and medication approval procedures as well as providing medication education and reconciliation by pharmacists is necessary [38]. Furthermore, improving the national capacity to maintain and distribute medical stockpiles in a timely manner, and pursuing a national strategic policy to incentivize local drug and PPE manufacturers to increase their investment to expand their industrial capacity to meet the local market needs are needed [39]. Additionally, NUPCO should increase the number of winners for any contract in the tendering process especially for essential drugs and medical supplies to minimize any unintended shortages in the future [40]. Moreover, the pricing of essential drugs by the Saudi regulatory bodies, such as the SFDA, should take into consideration the availability of these essential pharmaceuticals both locally and globally [10].

Although this is the first study to explore the impact of the COVID-19 pandemic on essential drugs and PPE in Saudi Arabia, several limitations must be acknowledged. First, this was a cross-sectional study and the list of potential participants was based on the available registered names in the Saudi Pharmaceutical Society database, which does not represent all the employees in the medical and pharmaceutical supply chain in Saudi Arabia. Therefore, we may not be able to generalize their responses to the entire country. The responses were self-reported and cannot be verified for accuracy. The response rate was approximately 60%; however, this was below the 80% desired threshold. In addition, the reasons behind shortages of drugs and PPE were not explored. Finally, this study focused mainly on essential medications, COVID-19 related therapies, and PPE. Therefore, information regarding the full impact of the pandemic upon drug shortages and medical equipment in general was not ascertained.

## 5. Conclusions

Shortages in essential medications and PPE are normal occurrences on an international scale. Therefore, it is expected for disruptions in the supply chain to occur when unexpected crises take place. Regardless, important lessons may be drawn and strategies created to curtail or lessen the impact of future unforeseen events. Policymakers in Saudi Arabia as well as the international community may benefit from the preparedness measures taken and opportunities missed mentioned, such as establishing an effective and reformed centralized procurement, developing and enforcing penalties to prevent price-gouging, mandating that data and dealings are maintained online and remain transparent, facilitating effective communication among all stakeholders, initiating and/or expanding local manufacturing practices, stockpiling essential drugs and equipment, and involving more pharmacists in the drug shortage mitigation processes.

## Figures and Tables

**Table 1:** Participants’ baseline characteristics (N = 103).

Characteristics	N (%)
**Age (yrs.)**	
25–34 yrs.	43 (41.7)
35–44 yrs.	40 (38.8)
45–54 yrs.	16 (15.5)
55–65 yrs.	4 (3.9)
**Gender**	
Male	67 (65)
Female	36 (35)
**Profession**	
Pharmacist	95 (92.2)
Physician	1 (0.97)
Engineer	1 (0.97)
Supply chain specialist	6 (5.83)
**Place of work**	
Ministry of defense- and aviation-affiliated healthcare institutions	25 (24.27)
Ministry of health-affiliated healthcare institutions	43 (41.75)
Ministry of interior-affiliated healthcare institutions	17 (16.50)
Ministry of national guard-affiliated healthcare institutions	5 (4.85)
University-affiliated hospitals	8 (7.77)
Private hospitals	5 (4.85)
**Geographic region**	
Riyadh	61 (59.22)
Makkah	13 (12.62)
AlMadinah	2 (1.94)
Asir	2 (1.94)
Albahah	2 (1.94)
Eastern province	19 (18.45)
Alqassim	1 (0.97)
Northern borders province	2 (1.94)
Jizan province	1 (0.97)
**Years of experience in supply chain**	
<1 year	13 (12.62)
1–2 years	10 (9.71)
2–4 yyears	6 (5.83)
4–6 years	11 (10.68)
6–8 years	17 (16.50)
>8 years	46 (44.66)

**Table 2:** List of drugs in short supply.

Name	N (%)
Tocilizumab	53 (51.45)
Hydroxychloroquine	44 (43.68)
Lopinavir/Ritonavir	35 (33.98)
Ribavirin	27 (26.21)
Enoxaparin	18 (17.47)
Interferon beta-1b	17 (16.50)
Dexamethasone	17 (16.50)
Cisatracurium besilate	14 (13.59)
Furosemide	11 (10.67)
Prednisolone	11 (10.67)
Methimazole	9 (8.73)
Hydrocortisone	9 (8.73)
Methylprednisolone	8 (7.76)
Levetiracetam	7 (6.79)
Piperacillin/Tazobactam	7 (6.79)
Vancomycin	4 (3.88)
Warfarin	3 (2.91)
Metformin	3 (2.91)
Ropivacaine	3 (2.91)
Pertuzumab	2 (1.94)
Trastuzumab	2 (1.94)
Bevacizumab	2 (1.94)

## Data Availability

The data are available upon reasonable request from the corresponding author.

## Appendix A

1. **Your age?**
  - 25–34 years
  - 35–44 years
  - 45–54 years
  - 55–65 years
  - >65 years
2. **Gender:**
  - Male.
  - Female.
3. **What is your profession?**
  - Pharmacist.
  - Physician.
  - Nurse.
  - Engineer.
  - Other:________________
4. **Your current position:**________________________
5. **How many years of experience do you have in the pharmaceutical supply chain/pharmaceutical purchasing or planning?**
  - None.
  - 1–2 years.
  - 2–4 years.
  - 4–6 years.
  - 6–8 years.
  - >8 years.
6. **Your hospital or healthcare institution is affiliated with which public or private entity:**_______________________
7. **The institution that you work in is located in which geographic region in the Kingdom?**
  - Riyadh.
  - Makkah.
  - AlMadinah.
  - Asir.
  - Albahah.
  - Eastern province.
  - Alqassim.
  - Northern borders province.
  - Jizan province.
8. **What was the approximate number of medications that you faced shortages with during the COVID-19 pandemic?**
  - None.
  - ≤10.
  - 10–20.
  - 21–30.
  - ≥30.
9. **Please mention the names of medications that your institution faced shortages with during the COVID-19 pandemic (A list of the World Health Organization (WHO) list of essential medicines is attached).**

___________________________________________________________________________________________________________________________________________________________________________________________________________________________________________________________________________________________________________________________________________________________________________________________________________________________________________________________________________________________________________________________________________________________________________________________________________________________________________________________________________________________________________________________________________________________________________________________________________________________________________________________________________________________________________________________________________________________________________________________________________________________________________________________________________________________________________________________________________________________________________________________________________________________________________________________________________________________________________________________________________________________________________________________________________________________________________________________________________________________________________________

10. **Please mention the names of the Personal Protective Equipment (PPE) that your institution faced shortages with during COVID-19 pandemic.**

______________________________________________________________________________________________________________________________________________________________________________________________________________________________________________________________________________________________________________________________________________________________________________________________________________________________________________________________________________________________________________________________________________________________________________________________________________________________________________________________________________________________________________________________________________________________________________________________________________________________________________________________________________________________________________________________________________________________________________________________________________________________________________________________________________________________________________________________________________________________________________________________________________________________________________________________________________________________________________________________________________________________________________________________________________________________________________________________________________________________________________________

11. **Kindly, estimate the difference in the acquisition cost of the Essential Medicines as shown in the WHO list of essential drugs pre- and post the COVID-19 pandemic.**
  - Same price.
  - 10–25%.
  - 25–50%.
  - 50–75%.
  - 75–100%.
  - >100%.
  - Other:____________
12. **Kindly, estimate the difference in the cost of PPE pre- and post the COVID-19 pandemic.**
  - Same price.
  - 10–25%.
  - 25–50%.
  - 50–75%.
  - 75–100%.
  - >100%.
  - Other:____________
13. **Did your institution place any direct purchase order for a drug or PPE during the COVID-19 pandemic due to drug or PPE shortages?**
  - Yes.
  - No.
